# Parkinson's Disease and Sleep/Wake Disturbances

**DOI:** 10.1155/2012/205471

**Published:** 2012-12-30

**Authors:** Todd J. Swick

**Affiliations:** ^1^University of Texas School of Medicine, Houston, TX, USA; ^2^Apnix Sleep Disorders Centers, Houston, TX, USA; ^3^The Sleep Center at North Cypress Medical Center, Cypress, TX, USA; ^4^Neurology and Sleep Medicine Consultants, 7500 San Felipe, Suite 525, Houston, TX 77063, USA

## Abstract

Parkinson's disease (PD) has traditionally been characterized by its cardinal motor symptoms of bradykinesia, rigidity, resting tremor, and postural instability. However, PD is increasingly being recognized as a multidimensional disease associated with myriad nonmotor symptoms including autonomic dysfunction, mood disorders, cognitive impairment, pain, gastrointestinal disturbance, impaired olfaction, psychosis, and sleep disorders. Sleep disturbances, which include sleep fragmentation, daytime somnolence, sleep-disordered breathing, restless legs syndrome (RLS), nightmares, and rapid eye movement (REM) sleep behavior disorder (RBD), are estimated to occur in 60% to 98% of patients with PD. For years nonmotor symptoms received little attention from clinicians and researchers, but now these symptoms are known to be significant predictors of morbidity in determining quality of life, costs of disease, and rates of institutionalization. A discussion of the clinical aspects, pathophysiology, evaluation techniques, and treatment options for the sleep disorders that are encountered with PD is presented.

## 1. Introduction

Parkinson's disease (PD) has traditionally been characterized by its cardinal motor symptoms of bradykinesia, rigidity, resting tremor, and postural instability. However, PD is increasingly being recognized as a multidimensional disease associated with myriad nonmotor symptoms including autonomic dysfunction, mood disorders, cognitive impairment, pain, gastrointestinal disturbance, impaired olfaction, psychosis, and sleep disorders [1, 2]. Sleep disturbances, which include sleep fragmentation, daytime somnolence, sleep-disordered breathing, restless legs syndrome (RLS), nightmares, and rapid eye movement (REM) sleep behavior disorder (RBD), are estimated to occur in 60% to 98% of patients with PD [3–6]. For years nonmotor symptoms received little attention from clinicians and researchers [7, 8], but now these symptoms are known to be significant predictors of morbidity in determining quality of life, costs of disease, and rates of institutionalization [9–15].

 James Parkinson, in his *Essay on the Shaking Palsy* published in 1817, noted that disturbed sleep, in addition to the motor symptoms, significantly affected many of the patients he studied [16]. He described “tremulous motion of the limbs occur during sleep, and augment until they awaken the patient, and frequently with much agitation and alarm.” In his description of “Case VI,” Parkinson wrote that the patient's attendants observed movements during sleep that increased until it awakened him: “when he always was in a state of agitation and alarm” [16]. This may be the first description of RBD, a condition that has been associated with PD; this nonmotor symptom can start years, if not decades, before the development of the classical clinical motor picture. By definition, RBD is characterized by loss of normal skeletal muscle atonia during REM sleep with prominent motor and behavioral activity and dreaming. 

However, RBD is not the only sleep-related nonmotor symptom that has been described as occurring as part of the spectrum of nonmotor symptoms associated with PD (and other synucleinopathies). These include excessive daytime sleepiness (EDS), RLS with or without periodic limb movements of sleep, insomnia (problems with sleep initiation, sleep maintenance, early morning awakenings, and the subjective feeling of nonrefreshing nocturnal sleep all leading to impaired daytime functioning), sleep-disordered breathing, and non-REM parasomnias (e.g., confusional arousals, somnambulism, somniloquy, sleep eating, and sexsomnia). 

## 2. Pathophysiology of Insomnia and Excessive Daytime Sleepiness

The pathophysiologic explanation of the motor symptoms of PD has been well characterized, albeit not without some controversy. The traditional hypothesis that Lewy bodies (LBs) and Lewy neurites (LNs), the characteristic intracellular proteinaceous inclusions of *α*-synuclein located in the soma and neuronal processes starting with degeneration of dopaminergic neurons within the substantia nigra (SN), has been brought under question by Braak et al. [17]. Their group proposed a six-stage progression of neuropathologic changes starting in the olfactory bulb, the anterior olfactory nucleus, and the dorsal motor nucleus of the glossopharyngeal and vagal nerves. The clinical correlate is olfactory dysfunction accounting for one of the first of the nonmotor symptoms. Stage 2 describes pathologic changes at the level of the medullary brainstem and then ascending to more rostral structures including the anteromedial temporal mesocortex and onto the neocortex. The SN is affected only in Stage 3, and the first motor symptoms are usually noted when the pathologic process has entered Stage 4 by which time most of the SN has already degenerated [17]. This sequence of pathologic changes may explain the earlier onset of many, if not all, of the nonmotor symptoms vis-à-vis the onset of the classic motor findings of PD or the other synucleinopathies [18].

As noted previously, sleep disturbances in PD are numerous and multifactorial. In broad terms, they can be divided into those disturbances that occur during the sleeping episode and those that occur during waking hours. The precise control of the circadian sleep/wake cycle and the ultradian REM/non-REM cycle is still not fully understood. However, there are generalizations that can be made and that have solid scientific support. 

 The ascending reticular activating system (A-RAS) plays a significant role in the maintenance of wakefulness. There are two paths, a dorsal pathway to the thalamic nuclei and a ventral pathway to the thalamus [19]. The mediating neurotransmitters include acetylcholine, serotonin, noradrenalin, dopamine, histamine, and hypocretin (orexin). 

The cholinergic neurons in the brainstem pontine laterodorsal tegmental (LDT) and the pedunculopontine tegmental nuclei (PPT) increase their firing rate approximately 1 minute before the first change to a desynchronized state on the electroencephalogram (EEG; REM onset) [20]. The LDT and PPT neurons send fibers via the dorsal pathway to the thalamus where they project to the thalamus and specifically to the intralaminar nuclei, thalamic relay nuclei, and reticular nucleus of the thalamus. This cholinergic input allows flow of information through the thalamus onto the cortex to promote cortical desynchronization (thalamocortical activation). The firing rates of the LDT/PPT neurons are sleep state-specific with high-frequency firing during wakefulness with decreased firing during Stages N1–N3 of non-REM sleep. During REM sleep, their activity abruptly becomes active again (thought to be secondary to release from monoaminergic inhibition) [21–25]. Mixed populations of noncholinergic (GABAergic) and cholinergic neurons in the basal forebrain (magnocellular preoptic nucleus in the substantia innominata, medial septal nucleus, and the nucleus of the diagonal band of Broca) send projections throughout the cortex, hippocampus, and amygdala. They have similar firing rates as the PPT and LDT nuclei, with the highest firing rates during wakefulness and REM sleep and the lowest during non-REM sleep [26]. 

The second branch of the A-RAS innervates the hypothalamus via the ventral route. These neurons are monoaminergic and include the noradrenergic locus coeruleus (LC), serotoninergic dorsal and median raphe nuclei, dopaminergic neurons of the ventrolateral periaqueductal gray matter (vlPAG), and the histaminergic neurons originating in the tuberomammillary nucleus [27–30]. These send fibers back to the basal forebrain, the ventral preoptic area (VPOA), and subsequently the entire cerebral cortex. These neurons also have state-specific firing rates; they collectively fire fastest during wakefulness, slow down during non-REM sleep, and nearly stop firing during REM sleep [25, 26]. These monoaminergic neurotransmitters are all associated with maintenance of wakefulness. 

The recent discovery of excitatory sleep/wake neuropeptides, hypocretin-1 and hypocretin-2, has added significant insight into our knowledge of the regulation of the sleep/wake state as well as offered etiologic explanations for the etiology of narcolepsy [31–34]. This may help explain some of the nonmotor aspects of PD (e.g., the excessive daytime hypersomnolence that frequently accompanies the disease).

Hcrt-1 and Hcrt-2 are produced from the same precursor, preprohypocretin (ppHcrt), by a small number of cells located in a paired set of nuclei in the lateral hypothalamus [35]. These neurons have diffuse projections throughout the central nervous system, including adjacent hypothalamic cell groups, the limbic system, the periaqueductal gray, dorsal raphe, and the lateral parabrachial nucleus. The Hcrt neurons received afferents from GABAergic, glutamatergic, and cholinergic neurons. 

 There are two known Hcrt receptors (HcrtR1 and HcrtR2), both of which are G-protein coupled receptors with excitatory effects [32] that interact with the two Hcrt moieties. HcrtR1 receptor is selective for Hcrt1, whereas Hcrt2 has a high affinity for both HcrtR1 and HcrtR2 receptors. This differing specificity supports the hypothesis that the two hypocretin neuropeptides perform different functions. It appears that Hcrt1 is responsible for maintenance of sleep and wake episodes, whereas Hcrt2 is involved in the maintenance of skeletal muscle tone while awake [36].

 The lateral hypothalamus contains several distinct cell groups, which contain neurons that produce neurotransmitters having differing effects on sleep/wake regulation. As previously discussed, the hypocretin neurons produce wake-active neuropeptides. In addition, there are neurons that contain melanin-concentrating hormone (MCH) that are codistributed with the Hcrt neurons; they are active during sleep, exhibiting slow firing during non-REM sleep, followed by a significant increase in firing during REM sleep. There is also evidence that these neurons actively inhibit the ascending monoaminergic systems to suppress wakefulness through feedback to the same monoaminergic cell groups that are activated by the Hcrt neurons [37].

Recently, another set of sleep-promoting neurons have been identified within the lateral hypothalamus that are GABAergic. GABA and galanin are inhibitory neurotransmitters that are found in the ventrolateral preoptic area (VLPO) and the median preoptic area (MnPO), both of which are instrumental in the onset of sleep [38, 39]. The sleep-active neurons in the VLPO fire fastest during non-REM sleep, with significant decrease in firing during REM sleep, and are silent during wakefulness [40–42]. The MnPO neurons begin to discharge just before non-REM sleep starts with persistence during both REM and non-REM sleep [25]. This has led to the hypothesis that MnPO neurons begin the process of sleep onset, whereas the VLPO neurons are necessary to maintain sleep (both non-REM and REM sleep) in conjunction with the MCH neurons. In addition, sleep maintenance is thought to be promoted by continuous firing of the VLPO and MnPO neurons along with the GABAergic cells within the lateral hypothalamus. They act to further inhibit the arousal system by inhibiting the Hcrt neurons that are adjacent to the lateral hypothalamus [43]. The significance of the knowledge of the neurochemistry and neuroanatomy of these sleep active centers is important in understanding possible mechanisms involved in the sleep pathology that accompanies most patients with PD.

### 2.1. REM Sleep Behavior Disorder (RBD) and Its Relationship to PD

The neural control of non-REM/REM sleep is particularly important for the understanding of the pathophysiologic process of RBD, one of the nonmotor symptoms of PD. The sublaterodorsal nucleus (SLD; also known as the subcoeruleus or peri-LC*α*), located just ventral to the LC, produces both GABA and glutamate. The cells in the SLD have a subgroup of projections to the medial medulla and the ventral horn of the spinal cord and are most active during REM sleep. Studies have shown that activation of the SLD region produces atonia and REM sleep-like EEG activity. There is also strong evidence that the SLD neurons are strongly inhibited by REM sleep-suppressing neurons in the midpons. These GABAergic cells are located in an arc starting in the vlPAG in the mesopontine tegmentum and extend out into the lateral pontine tegmentum (LPT). The vlPAG/LPT inhibits the SLD, and the SLD in turn inhibits the vlPAG, giving rise to the REM-on/REM-off flip-flop switch [25].

In the Braak schema, involvement of the pathologic process in PD affects the SLD, the magnocellular reticular formation (MCRF), and the perilocus coeruleus alpha structures as early as Stage 2 [17]. As noted, these cell structures play a significant role in not only regulating sleep/wake but also specifically modulating the REM/non-REM states. During REM sleep, there is generalized loss of muscle tone in all voluntary muscles with the exception of the diaphragm and the extraocular muscles (inability to act out dream mentation). In REM sleep without atonia there is loss of this protective mechanism. 

We normally exist in three states: wakefulness, non-REM sleep, and REM sleep. This schema is present in nearly all animal species. As such, animal studies have provided significant insight into the anatomy and neurochemistry involved in the orchestration of sleep onset/offset and REM/non-REM cycling as well as discerning the physiology and pathophysiology of REM sleep phenomena (i.e., dreaming, loss of muscle tone, and sympathetic activation).

As described earlier, RBD is characterized by persistence of muscle tone during REM sleep accompanied by motor/behavioral activity and dreaming. In the absence of any associated neurological disorder, it is called idiopathic RBD. When RBD occurs as a consequence of drugs or accompanies a separate identifiable neurologic disorder, such as narcolepsy or a neurodegenerative disorder, it is considered secondary or symptomatic RBD. 

It is possible to exhibit the electrophysiologic signature of RBD (i.e., REM sleep without atonia (RSWA)) on the overnight polysomnography (PSG) but never exhibit oneiric behavior. On the other hand, dream-enactment behavior can occur in other conditions such as untreated obstructive sleep apnea, sleep walking, and sleep terrors in adults, posttraumatic stress disorder, or as an effect of alcohol, drugs, or drug withdrawal. The American Academy of Sleep Medicine, publisher of the 2nd edition of *The International Classification of Sleep Disorders: Diagnostic & Coding Manual*, has stipulated that to diagnose RBD there has to be RSWA on the overnight PSG and either a history of injurious, potentially injurious, or disruptive behavior or abnormal sleep behavior during the PSG [44]. It needs to be noted that RSWA is not the same as RBD. Even though RSWA may represent a precursor to the development of clinical RBD, there is insufficient data in patients with RSWA to convincingly prove progression to the clinical syndrome [45]. 

Lesions of the subcoeruleus or perilocus coeruleus, MCRF, PPT, and LDT in cats and the SLD, which is equivalent to the subcoeruleus in rats, produced behavioral abnormalities consisting of complex motor activity during REM sleep consistent with the human syndrome of REM sleep without atonia [46, 47]. Boeve et al. have proposed that the SLD in humans with direct projections to the spinal interneurons is the final common pathway that causes active inhibition of skeletal muscle activity in REM sleep. They also propose that the indirect route can contribute to loss of muscle tone acting through the MCRF. Lesioning of the SLD results in reduced excitation of the MCRF thereby resulting in a decrease in the inhibition of spinal motor neurons directly or via spinal interneurons. It is not known if degeneration of the MCRF is sufficient to cause RBD in humans [45]. 

An interesting observation concerning the quality of motor behaviors and speech during the time of the motor/behavioral episodes of patients with RBD and PD has been reported. Using nocturnal videography during PSG and bed partner questionnaires, a surprising and significant improvement in the speed, strength, and fluidity of motor movements was observed or reported along with improvements in the quality of speech with evidence of improved articulation, loudness, and ability to be understood during the RBD episode. This phenomenon was also noted in patients with multiple system atrophy (MSA) during their episodes of RBD, but the degree of improvement was less [48]. It has been posited that movements during RBD originated in the motor cortex and descend through the normal pyramidal tracks bypassing the extrapyramidal system owing to the brainstem lesions that have disrupted the normal pontomedullary pathways that would ordinarily facilitate REM sleep atonia [49]. The association between RBD and clinically diagnosed PD was described by Schenck et al. in 1996, where there was a delayed emergence of parkinsonian symptoms in 38% of 29 men who were previously diagnosed with RBD (mean interval, 3.7 years) [50]. Since then, there have been numerous cases corroborating the association of RBD and PD, with some individuals developing RBD as long as 50 years before the onset of motor manifestations of PD [51]. However, the association between RBD and PD is not exclusive. RBD has been associated with dementia with Lewy bodies, pure autonomic failure (PAF), and other conditions that share a common pathology (e.g., Lewy body pathology, such as *α*-synuclein positive intracellular inclusions) [45, 52]. There also have been reports of RBD in patients with suspected progressive supranuclear palsy (PSP), spinocerebellar atrophy-type 3 (SCA-3), as well as a case of amyotrophic lateral sclerosis (ALS) and a case of Alzheimer's disease (AD) [53–56]. The frequency of RBD is between 33% and 60% in patients with PD [57], 50% and 80% in dementia with Lewy bodies (DLB) [58], and 80% and 95% in MSA [59]. Of note is that the nonsynucleinopathies PSP, SCA-3, and AD have tended to have RBD starting with or after the onset of motor symptoms of parkinsonism, whereas RBD typically starts years, if not decades, before the onset of cognitive and motor symptoms of PD, DLB, MSA, and PAF [45].

As previously noted, the temporal sequence of pathologic changes in patients with LB inclusions could explain why RBD precedes the motor symptoms of PD, with SLD impairment starting in Stage 2 of the Braak schema and the motor signs and symptoms not appearing until the pathologic process has progressed to Stages 3 and 4 with dementia appearing at Stages 4–6. This does not explain all the observations in the synucleinopathies, such as reasons that the majority of patients with PD do not exhibit dementia nor do patients with DLB exhibit oneiric behavior. One of the most frequent explanations for this discrepancy is that RBD may not start until there is sufficient degeneration of the relevant brainstem nuclei, and the motor symptoms of PD do not appear until there is at least 80% neuronal loss in the SN [45].

### 2.2. Excessive Daytime Sleepiness and PD

Excessive daytime sleepiness, defined as inappropriate and undesirable sleepiness during waking hours, is one of the most commonly reported sleep complaints in patients with PD, affecting between 15% and 50% of PD patients compared to age-matched controls depending on the population studied and the scales used to assess daytime sleepiness. The degree of EDS has been shown to be a key determinant of these patients' quality of life [1, 3, 60–63]. As a complaint, EDS is multifactorial—it can be caused by fragmented sleep resulting from primary sleep disorders such as obstructive sleep apnea, periodic limb movement disorder, narcolepsy, idiopathic hypersomnia, or behaviorally induced insufficient nocturnal sleep. Medications, pain syndromes, and numerous medical and psychiatric disorders also have been associated with significant EDS.

The severity of EDS in patients with PD has been shown to increase with disease severity and disease duration. There have been conflicting reports about the association of Hoehn and Yahr stage and the degree of EDS [4, 64]. It has been suggested that the degree of daytime sleepiness is related to the brainstem pathology of the PD itself, with direct effects on the wake/sleep (REM/non-REM-specific brainstem centers) and not on the nocturnal motor impairment, cognitive deficits (if any), or antiparkinsonian medications [65, 66]. However, dopaminergic agents have been implicated in causing sudden-onset sleep episodes (sleep attacks) and EDS in PD defined as “sudden, irresistible and overwhelming sleepiness that occurs without warning and is not preceded by being sleepy” [67]. It has been speculated that the EDS and episodes of sudden sleep attacks seen in some patients with PD are similar to that seen in patients with narcolepsy, a central hypersomnia characterized by severe EDS and intrusion of REM sleep phenomena during wakefulness (cataplexy, sleep paralysis, hallucinations, and sleep fragmentation). 

Narcolepsy with cataplexy is characterized by loss of hypocretin-containing cells in the lateral hypothalamus. It is thought to be due to an autoimmune process, but other etiologic factors have been reported to be associated with the loss of Hcrt neurons in the hypothalamus [31, 32]. The status of hypocretin levels in patients with PD has been controversial. Fronczek et al. [68] looked at three brain compartments to investigate the status of the hypocretin neurons in patients with PD. They measured Hcrt levels in postmortem ventricular cerebrospinal fluid, Hcrt concentrations as peptide fragments from the cerebral cortex, and counted the number of Hcrt neurons in the lateral hypothalamus. They found that Hcrt tissue concentrations were 40% lower in PD patients with only a 25% reduction in ventricular CSF levels. The reduction in the absolute number of Hcrt neurons was 50% lower than controls. Typical LB pathology was present in the perifornical hypothalamus, which established that the PD pathology was active in the affected region. Of note, LB inclusions were seen in only a minority of Hcrt neurons in these patients. This may explain why the reduction in Hcrt levels is not as complete as that seen in narcolepsy and why the clinical syndromes have overlapping features such as sleep-onset REM and RBD but no reports of cataplexy [69]. It has been postulated that patients with narcolepsy without cataplexy may have a reduced but not an absent number of Hcrt neurons in the hypothalamus.

Thannickal et al. reported that there was an increasing loss of Hcrt cells as PD progressed, and this was accompanied by a concomitant loss of MCH cells [70]. They rated the loss of cells across all Braak stages. At Stage 1, they found a 23% loss whereas at Stage 5 there was a 62% loss. MCH neural loss was lowest at Stage 1 at 12% and highest at Stage 5 at 74%. The losses were independent of disease duration. They posited that the sleepiness experienced by patients with PD is due to a combination of loss of Hcrt cells as well as changes that simultaneously occur in the dopaminergic, adrenergic, and serotonergic neurons—all of which have wakefulness—producing effects. 

In 1999, Frucht and colleagues reported on eight patients with PD who had abrupt onset of sleep episodes [71]. The “sleep attacks” occurred while the patients were driving and had taken either pramipexole (8 patients) or ropinirole (1 patient). The patient taking ropinirole had a prior sleep attack while taking pramipexole. Five of the eight had no warning (i.e., no prodromal sleepiness), and all episodes ceased with the withdrawal of the medications. Since the publication of this observation, there were numerous reports of other patients on pramipexole and ropinirole, as well as other dopaminergic agents including pergolide, bromocriptine, carbergoline, apomorphine, lisuride, piribedil, levodopa, tolcapone, and entacapone, who reported similar experiences [72–75]. 

 In 2002, Hobson et al. reported on the results of the Canadian Movement Disorders group survey of 638 “consecutive highly functional PD patients without dementia” to determine the frequency of and predictors for sudden-onset sleep, with particular emphasis on sleep attacks while driving [76]. Utilizing the Epworth Sleepiness Scale (ESS) and a scale designed for this particular study (the Inappropriate Sleep Composite Score), they found that EDS was present in 51% of the respondents. There was no significant difference in ESS scores for any of the dopaminergic agents that were used in terms of either composite scores or in the risk of falling asleep while driving. Sixteen patients (3.8%) had experienced at least one episode of sudden sleep onset (without warning) while driving. An ESS score of 7 or higher (scores of 9 or more are considered indicative of a greater propensity to fall asleep) was the greatest predictor of episodes of falling asleep behind the wheel. The conclusion of the study was that EDS is common in patients with PD who are independent and do not have dementia, but they felt that the sudden onset of sleep attacks was relatively uncommon.

Because of the limitations of survey results, Kaynak et al. studied 15 previously untreated patients with PSG/multiple sleep latency testing before and during treatment with DA. Before the initiation of DA therapy, there was no evidence of daytime sleepiness (both subjective and objective measures). The researchers found that UPDRS subset III scores were significantly improved with DA treatment; however, subjective and objective daytime sleepiness increased as measured by an increase in ESS scores and a decrease in the mean sleep onset latency on the multiple sleep latency test (MSLT) [77]. 

 Dopamine and its role in sleep/wake regulation remain unclear. From a pharmacologic standpoint, the release of dopamine (DA) and its reuptake inhibition by powerful stimulants such as amphetamines shows its wakefulness-promoting properties [78–80]. In fact, DA blockers have long been known for their sleep-inducing effects (e.g., chlorpromazine and haloperidol) [81]. It is now appreciated that DA has biphasic effects on wakefulness with low doses of DA-receptor agonists binding to autoinhibitory receptors on DA neurons, which in turn decrease DA signaling thus decreasing wakefulness with higher doses stimulating the wake-producing centers directly [65].

## 3. Other Associated Sleep Disturbances with PD

### 3.1. Restless Legs Syndrome and Periodic Limb Movements

Restless legs syndrome (RLS) is a common neurologic disorder that affects 4%–10% of the general population [82]. The clinical manifestations include uncomfortable or unpleasant sensations in the limbs (usually legs) that begin or worsen during periods of inactivity, are relieved by movement, and worsen during the night. In many, but not all patients there is an accompanying motor component of periodic limb movements (PLMs) that usually occur in sleep but can also occur during wakefulness (PLMW) [83]. PLMS may occur in association with RLS or independently. The pathophysiology is not clear-cut but it has been associated with iron deficiency and impairment of cerebral dopaminergic systems [84]. The robust response to dopaminergic agents for the treatment of RLS has been considered to support a relationship between RLS and PD. 

There have been numerous cross-sectional studies examining the frequency of RLS symptoms in patients with PD. The generally accepted frequency is 10%–20% association of RLS symptoms in patients with PD. In a 2011 study, Gjerstad et al. looked at 200 patients with early untreated PD and compared them to an appropriate community-based sample of controls [85]. They were unable to find a statistically significant association between RLS in patients with PD versus controls. They found that the patients with PD complained of leg motor restlessness (LMR) but did not have the “urge to move” that characterizes the sensory phenomenon in patients with RLS. Arnulf and Morgan in an editorial accompanying the Gjerstad article wondered if the LMR represents a forme fruste of RLS or is an unrelated phenomenon [86].

### 3.2. REM Sleep and Hallucinations

Treatment with dopaminergic agents has been associated with hallucinations in as many as 40% of PD patients. Fénelon et al. showed that three factors were independently predictive of visual hallucinations: severe cognitive impairment, duration of PD, and daytime sleepiness [87]. The association of RBD has been found to increase the risk of hallucinations by a factor of 3 [88]. It is postulated that the brainstem degeneration responsible for RBD is also responsible for the intrusions of dream mentation into wakefulness which are manifested as hallucinations [89–91]. PD hallucinations are best treated by the discontinuation of centrally acting anticholinergic agents, anxiolytics, antidepressants, and opiate pain medications. The use of the newer “atypical” antipsychotics has been shown to reduce the hallucinations without substantial worsening of the motor symptoms of PD, but their effects on RBD have not been studied [92]. 

### 3.3. Sleep-Disordered Breathing

The reported frequency of sleep-disordered breathing (SDB) in PD varies from 20% to 60% [65, 93, 94]. This is an unexpected association because the usual patient with PD is not obese, a factor that is a major factor in the development for SDB. Possible etiologic explanations include decrease in upper airway muscle tone because of degeneration of the brainstem serotoninergic neurons that innervate the muscles of the upper airway, deficient respiratory muscle coordination, or autonomic dysregulation [95–100]. In a cross-sectional survey, Chotinaiwattarakul et al. assessed the risk of SDB in patients with PD versus controls in a university-based movement disorders clinic. They identified a high risk of SDB in 49% of the patients with PD compared to 35% of controls. After adjustment for age, gender, and body mass index, patients with PD in comparison to controls showed a higher risk for SDB (OR = 2.81; 95% CI, 1.36–5.82). They also found that quality of life (physical component score) was significantly decreased in patients with PD at high risk for SDB. PD severity did not correlate with SDB risk, but patients with higher BMIs and ESS were at greater risk for the development of SDB [101]. 

## 4. Evaluation of Sleep Quality and Disturbances in the Parkinson's Disease Patient

Recognition and treatment of the nonmotor symptoms of PD are essential to the development of a holistic approach to a condition where there is significant variability in the time course and severity of all the components of the disease. The appearance of the majority of the nonmotor symptoms can precede by years, if not decades, the onset of the classic levodopa-responsive triad of motor symptoms (bradykinesia, hypokinesia, and rigidity). 

As noted, PD has significant negative effects on the overall quality of life with sleep disturbances such as insomnia, daytime sleepiness, and subjective nonrefreshing sleep causing frequent complaints. One of the first instruments devised to gauge the impact of sleep complaints on quality of life was the Pittsburgh Sleep Quality Index. First described in 1989, Buysse et al. created a self-rated questionnaire which sought to assess sleep quality and disturbances over a 1-month time frame [102]. The questions sought to determine the average amount and timing of the major sleep period, sleep latency, sleep disturbances, and subjective sleep quality. It specifically addressed such issues as sleep disordered breathing, dreaming, pain, use of sleep medications (prescription and OTC), jerking/kicking, and “restlessness.” It did not specifically address PD signs or symptoms other than on a general basis. 

 In 1987, the Unified Parkinson's Disease Rating Scale (UPDRS) was introduced to help codify and quantitate signs and symptoms of patients with PD for both clinical and research purposes [103]. There were four principal sections: (1) mentation, behavior, and mood; (2) activities of daily living; (3) motor examination; and (4) complications of therapy. Furthermore, there were two questions related to sleep and/or bedtime symptoms: the first dealt with “turning in bed and adjusting bed clothes” and the second was a yes or no question asking “Any sleep disturbances, such as insomnia or hypersomnolence?” 

With the growing awareness of the importance of sleep disorders within the context of PD, the Parkinson's Disease Sleep Scale (PDSS) was published in 2002 to help quantify sleep problems in patients with PD [104]. The PDSS was a visual analog scale that addressed 15 of the more commonly reported symptoms associated with sleep disturbances. The subject was asked to rate themselves on the following items using a visual analog scale with such ranges from “awful-excellent,” “always-never,” and “frequently-never”:

1. overall quality of sleep (item 1),
2. sleep onset and maintenance insomnia (items 2 and 3),
3. nocturnal restlessness (items 4 and 5),
4. nocturnal psychosis/oneiric mentation (items 6 and 7),
5. nocturia (items 8 and 9),
6. nocturnal motor symptoms (items 10–13),
7. sleep “refreshment” (item 14),
8. daytime dozing (item 15).

Also in 2002, another scale to assess sleep and sleepiness in PD was published, that is, Scales for Outcomes in Parkinson's disease-sleep (SCOPA-sleep) [105]. The scale was designed to evaluate both nighttime sleep and daytime sleepiness. The questionnaire, like the Pittsburgh Sleep Quality Index, sought to ascertain to what extent the individual had sleep/wake problems over the preceding month. Nighttime sleep items included queries concerning trouble falling asleep, nocturnal awakenings, early morning awakenings, and insufficient sleep. Daytime sleepiness questions were related to unexpected daytime or evening sleep episodes, unintentional sleep while sitting quietly, watching TV, or being engaged in conversations, difficulty staying awake during the day/evening, and did the individual consider falling asleep inappropriately as a “problem”?

In 2008, Martinez-Martin et al. evaluated the PDSS and the SCOPA-sleep scales by administering both scales to 187 consecutive PD patients [106]. They concluded that both scales provided valid and reliable means to evaluate sleep disorders in PD with the caveat that the PDSS provides a profile about potential causes of “bad sleep” but is insufficient for assessing daytime sleepiness issues, whereas the SCOPA-sleep addresses nocturnal sleep disorders and daytime sleepiness equally but neither explores the etiology. 

In 2008, a revised UPDRS (MDS-UPDRS) was published that included expanded questions concerning nonmotor symptoms of PD (i.e., hallucinations and psychosis, depressed and anxious moods, and apathy) and features of dopamine dysregulation syndrome (e.g., excessive gambling, excessive sexual drive, punding, spending, and shopping). There are two questions directly relating to sleep: (1) trouble going to sleep or staying asleep and how rested the individual is in the morning; and (2) does the individual have any trouble staying awake during the day? There are also questions about “turning over in bed,” gastrointestinal issues (constipation, chewing and swallowing, and drooling), autonomic problems (orthostasis), and a general question about fatigue [107].

As awareness of the impact of nonmotor symptoms on patients with PD increased, a Nonmotor Symptoms Scale (NMSS) was developed and validated through an international study in 2007. A 30-item scale was formulated containing nine dimensions: cardiovascular, sleep/fatigue, mood/cognition, perceptual problems, attention/memory, gastrointestinal, urinary, sexual function, and “miscellany” [108]. The nonmotor symptoms related to sleep disturbances included queries about falling asleep unintentionally, fatigue, or lack of energy, difficulties falling/staying asleep, and restlessness in legs [109].

Even with the PDSS, MDS-UPDRS, and NMSS, the scales were either inappropriate or insufficient in diagnosing specific sleep disorders in the context of PD. The main issues that needed addressing involved the specific types of insomnia, daytime episodes of sudden sleep onset, RBD, RLS, and sleep-disordered breathing [110, 111]. In 2010, a revised PDSS-2 was validated and released. The revised rating scale contained expanded nocturnal sleep conditions and disabilities that created a frequency measure scale with five categories to include previously overlooked conditions such as restless legs, akinesia, nocturnal pain, and sleep-disordered breathing. There are 15 questions about sleep and nocturnal disturbances that require the individual to rate themselves on a 0 (never) to 4 (very frequent) scale. Thus, the total score ranges from 0 (no disturbance) to 60 (maximal disturbance) [112].

It had been shown that fatigue in patients with PD significantly related to a substantial reduction in quality of life [113] and that the degree of fatigue was closely related to the quality and quantity of nighttime sleep. The PDSS-2 was able to address this factor without the need for additional scales. In addition, the PDSS-2 offers insights into the impact of the severity of the motor symptoms on sleep in three major areas. The first encompasses nocturnal motor symptoms such as akinesia, early morning dystonia, tremors while awake at night, limb movements during the night, restlessness, and questions relating to dream mentation (although there are no questions specifically referable to RBD). The second area focuses on PD-specific nocturnal nonmotor symptoms including hallucinations, confusional states, pain, muscle cramps, sleep-disordered breathing (e.g., snoring), and immobility. The third area involves sleep-specific disturbances including insomnia, sleep maintenance, quality of sleep as it relates to feeling tired or sleepy after the major sleep period, nocturia, and a general evaluation on the patient's perception of how well they slept over the past 7 nights. The issue of daytime sleepiness was purposefully left out, intending to concentrate on the nighttime features of patients with PD [112]. However, it should be noted that there is still a need for an instrument that reflects the entire range of sleep disturbances in patients who exhibit all of the Parkinson syndromes (e.g., MSA and progressive supranuclear palsy).

 The construction of the PDSS-2 has allowed researchers and clinicians to gauge the effects of new and existing drugs formulated to treat either motor and/or nonmotor symptoms of the PD patient on sleep. One such use has been the evaluation of rotigotine, a nonergot dopamine agonist, administered transdermally, which allows for continuous 24-hour administration in its effects on sleep as well as measuring the more traditional metrics for motor improvement. The RECOVER (Randomized Evaluation of the 24-hour COVerage: Efficacy of Rotigotine) was a multinational, double-blind, placebo-controlled trial of rotigotine in patients with PD who had unsatisfactory early morning motor symptom control [112]. RECOVER was the first large placebo-controlled trial in PD to assess nocturnal sleep disturbances as a coprimary outcome measure along with early morning motor function (using the UPDRS Part III) [114]. RECOVER also evaluated the effects of rotigotine on the nonmotor symptoms of PD using the Parkinson's disease NMSS as a secondary outcome measure [108, 109]. 

### 4.1. Evaluation of Sleep, Sleepiness, and Sleep Disruption in Patients with PD

One of the confounding issues when one considers the sleep disturbances in PD is the myriad scales for characterizing and measuring the multiple sleep abnormalities and assessing their degree of severity. There are factors that can elicit either or both excessive daytime sleepiness and nocturnal insomnia in the same individual. Sleep and wakefulness are physiologic states that normally transition from one to another without significant overlap. However, when there is continued disruption to either state, there develops a negative effect on both. In the broadest sense, the individual's degree of sleepiness depends on the interplay between two major factors: first, the time of the circadian cycle and, second, the length of time from the last sleep cycle [115]. The complaint of sleepiness can be normal or abnormal depending on the hour of day and the amount and/or quality of the previous night's sleep. In patients with sleep fragmentation on a nightly basis, a significant decrease in the total amount of sleep develops, and over a period of time this sleep loss amounts to a “sleep debt.” The net effect is an individual that is excessively sleepy [116, 117]. 

The concept of sleepiness can be broadly characterized by two components: there is the subjective perception of the need to sleep and there is the ability to transition from a state of wakefulness to a state of sleep [118]. The evaluation of sleep in patients with PD requires both subjective and objective inquiry and evaluation. One of the main stumbling blocks in developing scales and measurement instruments to accurately measure sleepiness and its physiologic effects is the wide array of terms that people use for their subjective awareness of the need to sleep. Patients can deny sleepiness but report fatigue, tiredness, or mental clouding. 

It is well known that the subjective complaint of sleepiness is variable in patients with similar sleep complaints, and the results of different screening tools when administered to the same patient typically have disparate findings. The ESS is useful as a subjective tool to assess the tendency of a person to doze in specific situations. However, it has less optimal correlation with more objective measurements such as the average sleep onset latency as determined by the MSLT or the ability to stay awake as measured by the maintenance of wakefulness test (MWT). 

One of the difficult issues in parsing the relationship between sleep, nocturnal sleep impairment, daytime hypersomnolence, and sudden onset of sleep attacks is that there is no one scale or set of methodologies that has been shown to be sensitive and specific enough to address such a broad range of signs and symptoms. Historically, sleep has been evaluated by history, scales, and objective measurements. As noted previously, a history needs to carefully distinguish what the patient means by their use of terms such as “fatigue,” “tiredness,” “exhaustion,” “drowsiness,” “lethargic,” and “groggy.” Psychiatric conditions, such as depression with apathy and lack of motivation, can be misinterpreted as sleepiness [110].

The overnight PSG is considered the “gold standard” for the evaluation of the nocturnal sleep structure and allows for the quantification of sleep disturbances [119]. The PSG is performed during the patient's major sleep period and measures EEG, eye movements (EOG), muscle activity (EMG), respiratory status with measurement of oral and nasal airflow/pressure, chest and abdominal respiratory efforts, and electrocardiography activity. Analysis of the raw data allows for the quantification of sleep onset, REM onset, and the amount and percentages of each of the different sleep stages and their distribution over the course of the night. The presence or absence of sleep-disordered breathing is identified as is the presence of abnormal limb movements. 

The PSG is a time-intensive and cost-intensive study. As such, other easier and less expensive methods have been developed to help with the assessment of sleep and its components. One such instrument is the actigraph. An actigraph is an instrument that is a small portable monitoring device that uses an internal accelerometer as a method for measuring rest/activity. It is typically attached to the wrist or ankle (it is configured like a wrist watch) and is worn for days or weeks collecting data based on preset algorithms (sampling ranges from seconds to minutes depending on its internal data storage system). The American Academy of Sleep Medicine has stated that actigraphy is not indicated for routine diagnosis, assessment of severity, or management of any of the sleep disorders. However, it may be useful as an aid in the evaluation of sleep-wake activity, particularly when looking at typical sleep/activity cycles in conditions such as insomnia, circadian-rhythm disorders, or medical and psychiatric conditions where it is difficult to keep an accurate sleep diary [120].

 Numerous scales have been developed that help identify and quantify different complaints within the sleep realm. One of the first of such instruments was the Stanford Sleep Scale (SSS) developed in 1972 [121]. This scale asks the respondent to rate themselves on a scale from 1 to 8, where 1 is “feeling active, vital, alert, or wide awake” to 8 “asleep” at that moment in time. This scale is often used just before the administration of an objective test such as the MSLT or MWT to compare the individual's perception of their degree of sleepiness to that found on the objective evaluation. 

The ESS questionnaire was developed [122] in 1991, and it consists of eight scenarios that represent different situations most people encounter or engage in during their daily lives. Respondents are asked to consider their likelihood of dozing (0 = no chance of dozing to 3 = high likelihood of dozing) in each of the separate situations even if they do not actually undertake them (such as lying down to rest in the afternoon or sitting in a car as a passenger for an hour without a break). They are asked to consider their answers, not on a day-to-day basis but rather over an extended period of time (e.g., 1 week). The highest score possible representing the greatest chance of falling asleep is 24 and the lowest score representing no chance of falling asleep is 0. Generally scores ≤9 are considered normal. There have been numerous studies showing adequate test-retest reliability [123]; however, there is poor correlation with MSLT results [124, 125].

 The MSLT is the most widely used objective test for the evaluation of sleepiness in clinical practice. Carskadon and Dement first described the MSLT in 1977 [126]. This test consists of five nap opportunities at 2-hour intervals starting approximately 2 hours after the conclusion of an overnight PSG. The time from lights out to the first epoch of sleep is noted for each nap as well as the presence or absence of REM sleep that occurs within 15 minutes of sleep onset for each of the naps. The patient is allowed up to 20 minutes to fall asleep. If no sleep occurs, the test is concluded 20 minutes after lights out. If sleep onset is within the 20-minute time span, the patient is allowed to sleep for an additional 15 minutes to observe for REM sleep. A minimum of 6 hours of sleep is required to be documented on the previous night's PSG because the MSLT has been shown to be sensitive to prior sleep deprivation [127]. The PSG also is used to exclude other sleep disorders such as obstructive sleep apnea syndrome or periodic limb movements as causes of daytime hypersomnolence. In 1986, a task force of the American Academy of Sleep Medicine published guidelines to help standardize the test in terms of procedures and findings. In 2005, the Practice Parameters Committee of the American Academy of Sleep Medicine published a revised set of guidelines for clinical use of the MSLT [128]. 

 The interpretation of the MSLT as per the previously mentioned guidelines establishes that a mean sleep onset latency of <5 minutes (averaged over the 5 naps) indicates that the subject is pathologically sleepy, whereas the *International Classification of Sleep Disorders: Diagnostic & Coding Manual*, 2nd edition, delineates a mean sleep latency of ≤8 minutes as one of two MSLT criteria necessary and sufficient for the diagnosis of narcolepsy [44]. A value of >10 minutes is considered to be statistically normal. The presence of sleep onset REM periods (SOREMPs) on two or more naps is considered abnormal and consistent with narcolepsy [128]. It needs to be noted that SOREMPs can also be seen in 7% of untreated patients with sleep apnea and also have been reported in patients with PD and MSA [129–131].

 The MWT, a derivative of the MSLT, is used to measure a subject's ability to stay awake. The patient is generally seated in bed or in such a position that supports the back and head in a quiet, dimly lit room. The patient is instructed to remain awake and alert as long as possible. Each trial is terminated after three epochs of Stage 1 or one epoch of any other stage of sleep. If there is no sleep onset, the test is ended after 40 minutes. Typically, four 40-minute trials are performed [128]. The MWT is useful as an objective measure of one's ability to stay awake when such ability is required for public safety reasons. This is particularly important to measure the effectiveness of treatment for conditions such as obstructive sleep apnea (OSA) in people who drive buses, operate trains, or fly airplanes. The US Federal Aviation Authority has mandated the use of the MWT for pilots who are being treated for OSA before they can resume flying [128]. 

## 5. Treatment of Sleep Disorders in PD

As is the case with most sleep disorders, treatment needs to be directed towards targeting the underlying mechanism of the major sleep complaint, that is, improving the sleep at night and/or improving daytime wakefulness. It is necessary to carefully evaluate the sleep patterns and the presence of abnormal movements and/or behavior and their frequencies during the night. This information should be obtained from the patient's bed partner (if available). Careful note of the number of awakenings and characterization of the daytime sleepiness, for example, does the individual experience sudden sleep attacks, excessive drowsiness that interferes with daytime functioning, and does the individual take naps (how often and for how long?) need to be ascertained. In addition to obtaining a general medical history it is important to inquire about all comorbid conditions that can impact sleep-wake regulation and perform a thorough review of the medications that the patient is taking for their PD and any other conditions including over-the-counter drugs. If they are taking dopamine agonists (DA) and experience EDS they should be warned not to drive or engage in hazardous activities. Downtitration or discontinuation (if tolerated) of a DA often helps [132]. However, further upregulation may be effective if the patient is still undertreated and/or has recently started treatment.

 As noted previously, the RECOVER study demonstrated significantly greater improvement with the rotigotine patch (nonergot 24 hr DA delivery) compared to placebo in terms of mean UPDRS Part III and PDSS-2 scores from baseline to end of treatment [114]. 

Another problem with the long-term use of DA drugs is that these agents have deleterious effects on sleep when administered at bedtime with the development of sleep disruption (DA in high doses have been considered stimulants) [133]. Recently there have been numerous reports of the development of dopamine dysregulation syndrome affecting up to 11% of patients with PD characterized by uncontrollable drives including hyperphagia, hypersexuality, punding, gambling, and shopping in patients taking DA agents in all dose ranges [134]. 

Many of the NMS that have been previously discussed have a direct impact on the sleep/wake state including RBD, RLS, SDB, nocturnal hallucinations, and PLMDs. Pharmacologic therapy for RBD has been based on case reports because there have not been any double-blind placebo-controlled trials of sufficient numbers of patients to allow for clear-cut preferences. However, common clinical practice papers have been published describing the use of clonazepam as the treatment of choice for the management of RBD [135]. Other drugs have also been reported to decrease the frequency and/or severity of RBD. These include melatonin, levodopa, pramipexole, carbamazepine, donepezil, galantamine, triazolam, clozapine, and quetiapine [136–142]. There are several pharmacologic agents that can induce or aggravate the symptoms of RBD. These include monoamine oxidase inhibitors, tricyclic antidepressants, and selective serotonin reuptake inhibitors (SSRIs). The most common agents that have been implicated are fluoxetine and mirtazapine [135]. A PSG study looking at patients taking SSRIs found an increase in electromyogram (EMG) activity during tonic submental REM sleep compared with control subjects [143].

Idiopathic RLS is generally treated with DA; however within the context of PD patients who are already on DA, treatment with agents such as gabapentin enacarbil or opiates has also been used [144]. It should be noted that caffeine, alcohol, central acting antihistamines, dopamine antagonists, tricyclic antidepressants and serotoninergic reuptake inhibitors can exacerbate RLS [145]. Iron deficiency can also contribute to RLS symptoms and replacement therapy should be considered if the serum ferritin is <50 *μ*g/L [146]. 

Nasal continuous positive airway pressure (CPAP) is the “gold-standard” for the treatment of obstructive sleep apnea. Generally treatment should be started on patients with an apnea/hypopnea index of 15 or more per hour of sleep as determined during overnight polysomnography [93]. 

The use of melatonin in PD patients with sleep maintenance difficulties has been shown to be effective in terms of improving subjective assessments of sleep quantity and daytime sleepiness [147]. The use of short acting hypnotics such as the nonbenzodiazepine agonists (zolpidem, eszopiclone, and zaleplon) have been used but they carry significant risks of psychomotor impairment during the night if the patient awakens and ambulates. The association of these nonbenzodiazepines with confusional arousals/automatisms also needs to be considered [148, 149]. 

As previously noted, EDS is an extremely common complaint of PD patients. In terms of treatment, one needs to try to reduce any medications that are sedating, but frequently manipulation of the doses of these drugs does not produce any significant reduction in the EDS. In some cases the addition of a psychostimulant during the day is recommended. Modafinil, a medication that is FDA approved for the EDS of narcolepsy and sleepiness associated with OSA and sleep-work shift disorder, has been demonstrated to be well tolerated in PD patients but, its alerting effect is not robust (<33% of patients responded) [150]. In a small trial, nocturnally administered sodium oxybate, a drug that is used to treat both EDS and cataplexy in narcoleptic patients, increased slow-wave sleep as a percentage of total sleep time and improved subjective nighttime and daytime sleep complaints in patients with PD [151]. 
